# A direct effect of perception on action when grasping a cup

**DOI:** 10.1038/s41598-017-18591-5

**Published:** 2018-01-09

**Authors:** E. Rounis, V. van Polanen, M. Davare

**Affiliations:** 1 Nuffield Department of Clinical Neurosciences, University of Oxford, Oxford, United Kingdom; 2 Motor Control Laboratory, Movement Control and Neuroplasticity Research Group, Biomedical Sciences Group, Department of Movement Sciences, KU Leuven, Belgium; 3 Sobell Department of Motor Neuroscience and Movement Disorders, Institute of Neurology, University College London, London, United Kingdom

## Abstract

Affordances represent features of an object that trigger specific actions. Here we tested whether the presence and orientation of a handle on a cup could bias grasping movements towards it in conditions where subjects were explicitly told to ignore the handle. We quantified the grip aperture profile of twelve healthy participants instructed to grasp a cup from its body while it either had no handle, a handle pointing towards, or away from the grasping hand (3 ‘move’ conditions, with large grip aperture). To ensure the smaller grip aperture afforded by the handle was implicitly processed, we interspersed trials in which participants had to grasp the cup from its handle or a handle not attached to a cup with a small grip aperture. We found that grip aperture was smaller in the presence of a handle in the ‘move’ conditions, independently of its orientation. Our finding, of an effect of the handle during the execution of a grasp action, extends previous evidence of such an influence measured during motor preparation using simple reaction times. It suggests that the specific action elicited by an object’s attribute can affect movement performance in a sustained manner throughout movement execution.

## Introduction

The concept of ‘affordance’ was introduced by Gibson^1^ to describe how features of objects (such as their physical properties) may be linked to a functional goal and the physical properties of an actor. Several behavioural studies have demonstrated that affordances modulate human movement performance, independent of the intention to act^2–10^. For example, Tucker and Ellis^2^ instructed participants to make finger presses with their right or left hand in response to the orientation (up or down) of a handled object on a screen. They observed faster response times if the orientation of the handle on an object was compatible with the responding hand, reflecting the ‘automatic’ activation of an action to grasp the handle. Further studies have demonstrated that object features may ‘automatically’ trigger components of specific actions, such as reaching and grasping^3–5^.

Nevertheless, the exact nature of affordance ‘effects’ remains elusive. Some authors have argued they represent spatial compatibility effects reported in other contexts^6–9^.

A study by Tipper *et al*.^10^, described attentional effects using distractor-target combinations which demonstrated spatial- and arm-specific biases during reaching. Later studies investigating distractor-target perturbations extended this work by providing evidence of similar processes in prehension^11–14^. It is noteworthy that even though the behavioural consequences of affordances may be related to attentional mechanisms, these effects are behaviourally separable to visuospatial attention. Indeed, they are present even when the object is irrelevant to the task^2^ and when visuospatial attention is directed away from the object^15–17^.

Further work on the nature of affordance effects suggests that affordances can trigger motor representations^18,19^. Studies in non-human primates have identified areas in the dorsal stream, which are responsible for selecting the appropriate motor programs for object-directed actions^20–22^. A ‘dorso-medial’ network, comprising areas in the posteromedial portion of the intraparietal sulcus and dorsal premotor cortex, implicated in on-line reaching and action selection, interacts with a ‘dorso-lateral’ network comprising area F5 and anterior intraparietal regions (AIP), implicated in grasp selection^21–23^. Recent studies suggest that computations for reach and grasp actions might be integrated^24^. The degree of interaction between them depends on task demands, the degree of online control and visual feedback required by an action^25,26^. Human homologues of these areas have been demonstrated in functional neuroimaging studies^27–30^. Additionally, motor representations triggered by affordances have been elicited with techniques such as TMS^31,32^ and EEG^33–35^. A study by Buccino *et al*.^31^ demonstrated greater motor excitability in the affording effector specifically when participants were presented with images of handled objects in which the handle was intact (and could therefore be grasped by the tested hand) and not in images of these objects in which the handle was ‘broken’.

The studies mentioned above have described ‘affordance’ effects mostly during action planning. However, the use of behavioural measures, such as reaction times, to measure these effects, has been criticised for failing to represent the full nature of the action triggered by objects^6^. One way to remedy this would be to study affordance effects during action *execution*.

This was done in an original study derived from the neuropsychology literature^36^. A patient with alien-limb syndrome due to cortico-basal degeneration (CBD) was asked to pick a cup on the right or left side of the table using the hand that was on the same side of the table as the object, irrespective of the orientation of its handle. When the cup was on the left, and its handle oriented to the right, the patient tended not to grasp the object with her left hand (the required response) but rather grasped it with her right hand, i.e. the grasp action being cued by the orientation of the object in relation to the patient’s preferred hand. This grasp action preference was felt to demonstrate an affordance of the handle on the cup, and was specific to this action as it was not elicited when the patient was asked to point to the cup^36,37^.

Based on these early findings we sought to investigate whether affordances, whose effects have mainly been described on movement planning, could elicit movement representations during the execution phase of a reach-to-grasp action to an object, in healthy human volunteers. We used quantifiable kinematic measures of movement, namely the grip aperture (i.e. the distance between thumb and index finger) to investigate the effect of the presence and orientation of a handle on a cup, whilst participants were asked to grasp the object from its rim in order to ‘move’ it to another location.

**Figure 1:**
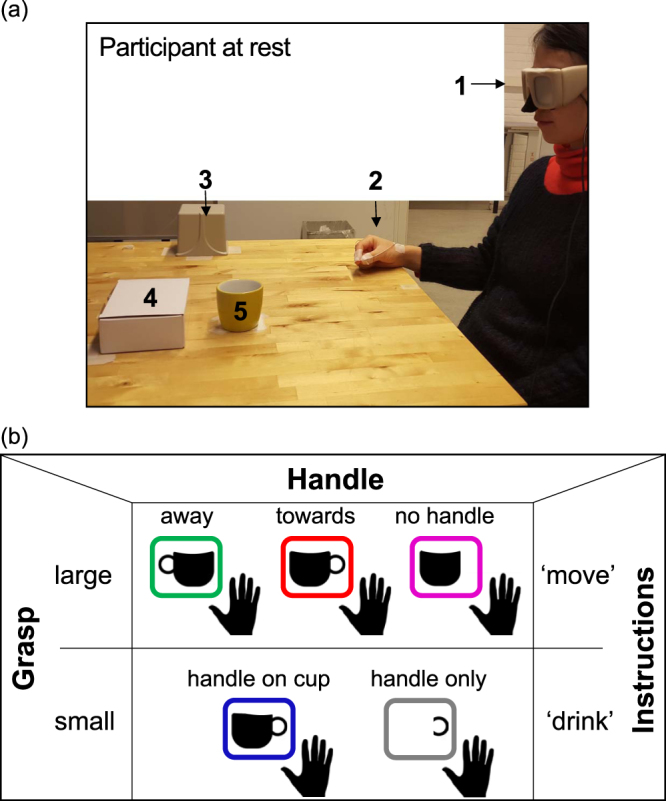
Grasping a cup to ‘move’ or to ‘drink’ from it. (**a**) Shows a picture of the set-up: 1) PLATO glasses (closed in this case), 2) starting position of the hand (with thumb and index finger pinched and position sensors in place), 3) TrakSTAR transmitter device, 4) platform for placing cup in the ‘move’ conditions, 5) cup with the handle oriented towards the grasping hand. (**b**) Demonstrates task conditions: 12 Participants were presented with one of three objects: a cup with a handle (that was either pointing ‘towards’ or ‘away’ from the grasping hand), a cup with no handle and a ‘handle only’. They were either instructed to grasp a cup to ‘move’ it; or to pincer grip the handle to ‘drink’ from it. The former required them to grasp the cup from its top end and move it from a central position on a table to a box that was adjacent and to the back of the object. The latter instruction was only provided for the cup with the handle oriented toward the grasping hand (‘handle on cup’) or with the ‘handle only’ that had the same dimension as the handle of the cup. Instructions were provided verbally at the same time as the object was presented by opening of PLATO-occlusion glasses. Each of the 5 conditions was presented randomly and repeated over 20 trials.

We hypothesised that grip apertures would be smaller in the presence of a handle on the cup, particularly if this was oriented in the direction of the grasping hand, as in previous studies investigating the role of affordances on actions^2–6^. However, there was a concern that due to the need for many repetitions of these trials, any affordance effect of the handle on the cup would not be detected if participants were not engaged in grasps targeted to the handle of the cup as well. To this end, trials with a ‘move’ instruction were interspersed with ones where participants had to grasp the handle of the cup, in order to ‘drink’ from it, to ensure the *small* precision grip afforded by the handle was implicitly processed. An additional ‘drink’ condition was included, which involved grasping a handle on its own whose purpose was to investigate affordance effects of the body of the cup on the handle in the drink conditions^17^ (Fig. 1b, see also videos in supplementary material). This generated a total of five conditions: two for the ‘drink’ actions, and three for the ‘move’ actions: one with the handle directed towards the grasping hand, one with the handle away, and one with no handle.

Participants were instructed to hold their index and thumb fingers together (in a pincer grip) at the start of every movement before making the grasp action required for the task: namely a small precision grip to grasp the handle when instructed to ‘drink’, or a larger precision grip to grasp the cup from its rim when instructed to ‘move’ it. Each movement was self-paced and the start of the movement was indicated by the opening of PLATO-occlusion spectacles (see Methods).

In addition to investigating affordance effects at the time of maximum grip aperture prior to grasping the object, we examined the time course of these effects throughout the movement execution phase by quantifying the grip aperture profile in 10% steps from movement onset until maximum grip aperture^38^.

## Results

**Figure 2:**
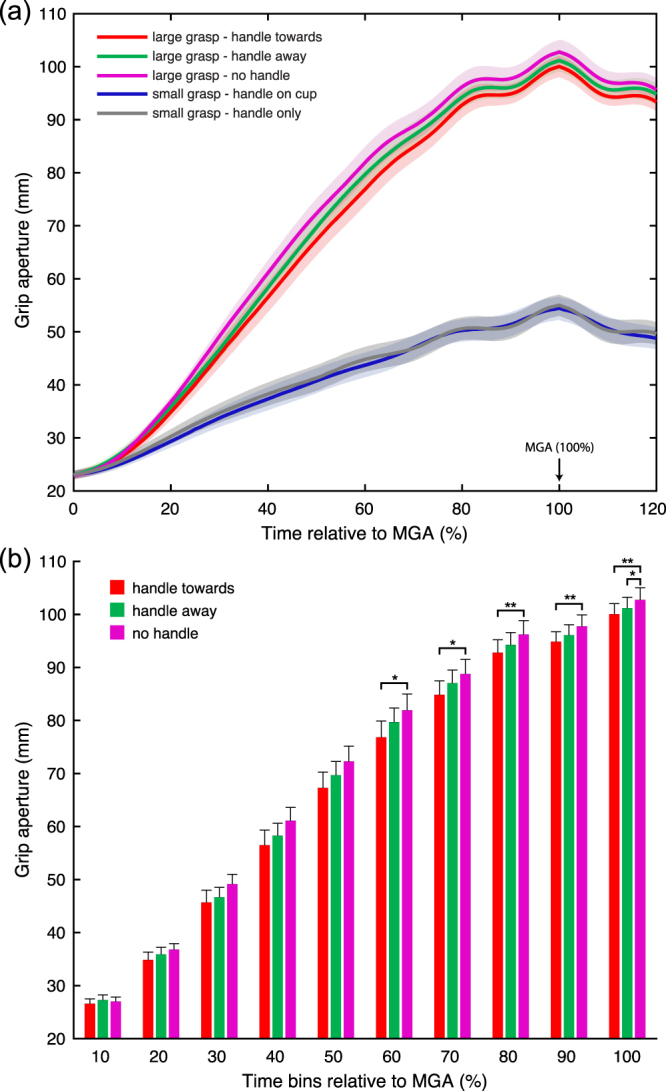
Grip Apertures during transport to grasp the object. (**a**) Shows the grip apertures averaged over participants for each of the 5 conditions from start until just after MGA (100%, line) is reached. Participants started with their hand pinched. Their grip apertures were wider in the ‘move’ compared to the ‘drink’ conditions. In the former conditions, we observed significant modulation in grip aperture relating to the presence of a handle, even though the dimensions of the cup that was moved were identical. The MGA was largest in the move conditions when the cup had no handle. It was significantly reduced when the cup had a handle oriented away and when this was oriented toward the grasping hand. The grip apertures for the two ‘drink’ conditions, namely with a ‘handle on cup’ or a ‘handle only’ showed no significant modulation. (**b**) Represents the grip aperture histograms for each of the 3 move conditions. The data has been grouped in 10% of MGA time points, demonstrating significant differences in grip aperture between conditions over time (handle toward vs no handle occurring at 60% until 100% of MGA, handle away vs no handle occurring at MGA, see results section). *p =  < 0.05, **p < 0.001.

**Figure 3:**
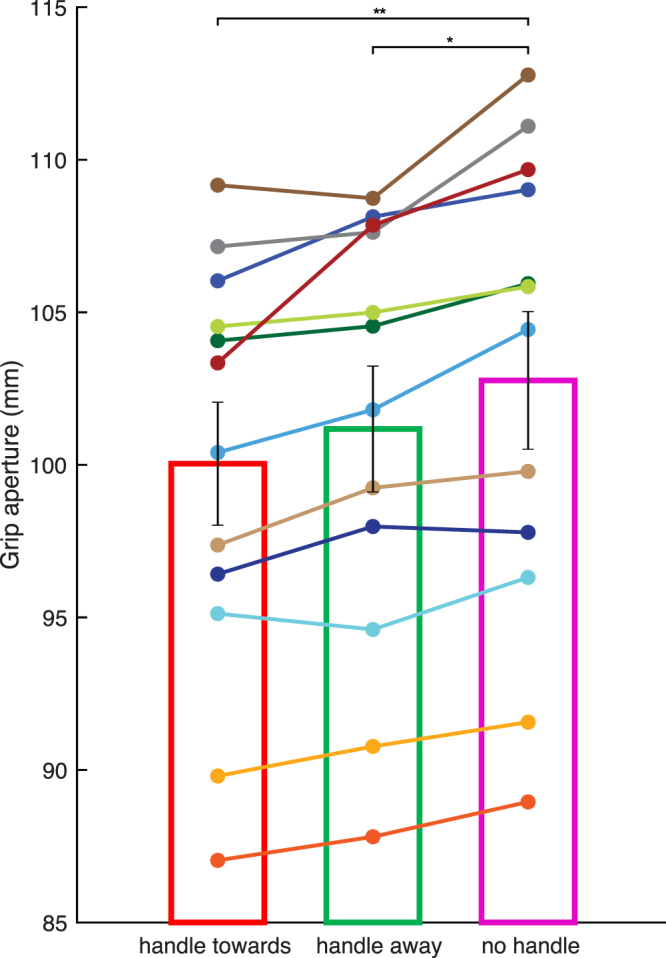
Maximum grip aperture differences between subjects across the 3 ‘move’ conditions. This figure shows the modulation in maximum grip aperture (MGA) in the three ‘move’ conditions, according to presence and orientation of a handle on the cup. The three bars represent the MGA in the condition with a handle pointing towards the grasping hand, away from it and no handle, respectively. Each line represents a single subject and bars represent averages across subjects with error bars indicating standard errors, demonstrating consistent effects across participants. *p =  < 0.05, **p < 0.001.

Kinematic data from a total of 12 participants were analysed for grip apertures relating to the five conditions of our task, leading to a large precision grip (in the three ‘move’ conditions) and a small precision grip (in the two ‘drink’ conditions). The results of these analyses are outlined below and in Figs 2 and 3.

### Errors

The error rates were analysed using a one-way repeated-measures analysis of variance (ANOVA) with five conditions (three ‘move’ and two ‘drink’ conditions) entered for each participant to identify whether error rates were different between conditions. There were 2.9% of errors across all trials, which were excluded from the overall analysis. These included errors caused by the experimenter (e.g. positioning the wrong cup, 0.33%), or errors made by the participant. In the latter category, there were errors in failing to initiate the movement with a pincer grip – leaving an open gap between the thumb and the index finger (0.25%) – these trials were excluded due to difficulty estimating the maximum grip aperture. The remaining errors (1.81%) related to errors made by participants in the different trial types (e.g. performing a small precision grip instead of a large one when asked to ‘move’ the cup or vice versa when asked to ‘drink’). The ANOVA revealed no significant main effect [F(1,4) = 1.14; MSE = 0.39; *η* ^2^ = 0.35; p = 0.09], suggesting these errors were distributed in approximately equal numbers across all conditions.

### Grip apertures

The maximum grip apertures (MGA) for each of the five conditions were entered into another one-way repeated-measures ANOVA with the factor ‘HANDLE’ (‘*towards*’, ‘*away*’, ‘*no handle*’ in the three ‘move’ conditions, ‘*handle only*’ and ‘*handle on cup*’ in the two ‘drink’ conditions). This ANOVA, with a Greenhouse-Geisser correction, showed significant differences in MGA between all the handle conditions [F(1.29, 14.15) = 379.13; MSE = 64.16; *η* ^2^ = 0.97; p < 0.0001], as illustrated in Figs 2 and 3.

We describe changes in grip aperture in the ‘move’ and ‘drink’ conditions separately, in the paragraphs below. In the post-hoc t-tests described below, we applied a Bonferroni correction for multiple comparisons across all 5 conditions (see Methods).

### Grip apertures in the ‘move’ conditions

The MGAs for ‘move’ conditions were all significantly larger than those for the ‘drink’ conditions, and were also significantly different from each other (Figs 2 and 3).

The smallest MGAs in the ‘move’ group were the ones with a handle. Specifically, the ‘move’ condition with the handle pointing ‘towards’ the grasping hand (Mean = 100.04 mm, SEM = 2.01 mm), led to similar grip apertures to the one with the handle pointing ‘away’ (Mean = 101.18 mm, SEM = 2.06 mm; t(11) = −2.9, p = 0.14). Both grip apertures were significantly smaller compared to the grip aperture when there was ‘no handle’ on the cup (Mean = 102.77 mm, SEM = 2.25 mm; towards versus no handle: t(11) = −6.2, p = 0.00069; away versus no handle: t(11) = −4.4, p = 0.0099). These results were consistent across participants and are outlined in Fig. 3.

### Effects at earlier time points

Figure 2a shows the grip aperture profile averaged across participants in each task condition from the start of movement to the time just after participants reached their MGA. Traces were normalised relative to the time subjects reached MGA (100%).

In order to identify whether the MGA modulation induced by the handle in the ‘move’ conditions was also present earlier during movement execution, we analysed the grip aperture profile at several time points leading to the MGA time. Intervals of 10% were taken from 10-100% of time to MGA and analysed in a 10 (%MGA step) × 3 (HANDLE) repeated measures ANOVA. The Greenhouse-Geisser corrected main effects of ‘%MGA step’ was significant [F(1.83,20.1) = 388.43; MSE = 319.9; η^2^ = 0.97; p < 0.0001]; as was the main effect of ‘HANDLE’ [F(2,22) = 10.50; MSE = 31.89; η^2^ = 0.49; p = 0.001], and the interaction between the two [F(3.04,33.5) = 3.56; MSE = 11.42; η^2^ = 0.24; p = 0.024]. The differences in MGAs between the three ‘move’ conditions were present at early stages. Post-hoc comparisons with Bonferroni correction revealed the first difference in grip aperture to emerge between the ‘handle toward’ and ‘no handle’ conditions when participants had reached 60% of their MGA before grasping the object. This effect persisted until participants reached MGA. The difference in grip aperture between the ‘handle away’ and the ‘no handle’ conditions appeared at a later stage when participants reached MGA. The details of these differences (including statistical significance) throughout the reaching phase are highlighted in Table 1 and in Fig. 2b.

**Table 1:** Changes in grip aperture in the move conditions measured in 10% MGA steps (Bonferroni corrected pairwise comparisons in the three ‘move’ conditions for each step).

% MGA	Conditions	T value (df (11))	Sig (2-tailed)
10	handle toward vs handle away	−1.74	3.27
	handle toward vs no handle	−1.19	7.71
	handle away vs no handle	0.825	12.81
20	handle toward vs handle away	−1.14	8.31
	handle toward vs no handle	−2.95	0.39
	handle away vs no handle	−1.19	7.77
30	handle toward vs handle away	−0.76	13.89
	handle toward vs no handle	−3.09	0.3
	handle away vs no handle	−2.41	1.02
40	handle toward vs handle away	−1.24	7.26
	handle toward vs no handle	−3.11	0.3
	handle away vs no handle	−2.2	1.5
50	handle toward vs handle away	−1.98	2.19
	handle toward vs no handle	−3.75	0.09
	handle away vs no handle	−2.1	1.77
60	handle toward vs handle away	−2.75	0.57
	**handle toward vs no handle**	**−4.35**	**0.03**
	handle away vs no handle	−2.09	1.8
70	handle toward vs handle away	−3.07	0.33
	**handle toward vs no handle**	**−4.35**	**0.03**
	handle away vs no handle	−2.16	1.62
80	handle toward vs handle away	−2.33	1.2
	**handle toward vs no handle**	**−5.27**	** < 0.001**
	handle away vs no handle	−2.98	0.36
90	handle toward vs handle away	−2.79	0.51
	**handle toward vs no handle**	**−6.07**	** < 0.001**
	handle away vs no handle	−3.85	0.09
100	handle toward vs handle away	−2.92	0.42
	**handle toward vs no handle**	**−6.19**	** < 0.001**
	**handle away vs no handle**	**−4.47**	**0.03**

### Grip apertures in the ‘drink’ conditions

As mentioned in the introduction, ‘drink’ trials requiring a small precision grip were interspersed with the ‘move’ trials, requiring a small precision grip. The purposes of these trials were 1) to ensure the implicit manipulation afforded by the handle in the ‘move’ trials, as in previous studies investigating affordances^2–6^ and 2) to investigate whether the large precision grip prompted by the cup led to larger grip apertures when preparing a small precision grip towards the handle. Two conditions, involving a small precision grip directed to the object, were generated to enable this: one involving grasping the handle of the cup, which in this case was always oriented in the same direction as the grasping hand (‘handle on cup’), and one involving grasping a handle on its own, with no cup (‘handle only’). Both conditions were preceded by the instruction to ‘drink’, indicating that a small precision grip was to be used.

A paired samples t-test comparing the two ‘drink’ conditions revealed no significant differences in MGA between the ‘handle only’ (Mean = 54.48 mm, SEM = 2.17 mm) and ‘handle on cup’ conditions (Mean = 55.03 mm, SEM = 1.99 mm; t(11) = −0.47, p > 0.1). For this reason, we did not analyse changes in grip aperture at earlier time points.

### Influence of the previous trial on grip aperture

We investigated whether there was an influence of the grip aperture performed in the previous trial on the affordance effects induced by the handle, described above. To this end, we performed a further two-way repeated-measures ANOVA with factors: ‘grip type in the previous trial’ (large (in the ‘move’ conditions) or small (in the ‘drink’ conditions) precision grip) and ‘HANDLE’ (5 conditions, as above).

This ANOVA again revealed a main effect of ‘HANDLE’ [F(1.27,13.9) = 373.32; MSE = 133.28; η^2^ = 0.97; p < 0.0001], which was mostly driven by differences between large and smaller grip apertures for drink and move conditions (see supplemental material 2). The main effect of ‘grip type in the previous trial’ [F(1,11) = 0.06; MSE = 5.83; η^2^ = 0.005; p = 0.81], and the interaction between the two [F(4,44) = 2.06; MSE = 2.68; η^2^ = 0.16; p = 0.15] were not significant. This suggests that the effects we observed were not related to a carry-over effect or priming influence of the previous trial on the next one, but were genuinely relating to the influence of the presence and orientation of the handle on the cup.

### Other performance measures influenced by affordances

We investigated the influence of the handle and previous grip on the speed of movement preparation and movement execution, using reaction and movement times, respectively (shown in Table 2).

**Table 2:** Reaction times (RT) and movement times (MT) for each condition.

Move			Drink		
HANDLE	Toward	Away	No handle	Handle on cup	Handle only
RT (s) and S.E.M.	0.67 (0.02)	0.66 (0.03)	0.63 (0.02)	0.68 (0.02)	0.69 (0.02)
MT (s) and S.E.M.	0.68 (0.02)	0.68 (0.02)	0.67 (0.02)	0.69 (0.02)	0.71 (0.03)

Reaction times (RTs), defined as the time between the opening of the glasses and movement onset, were entered in a further one-way ANOVA with five conditions. This revealed a significant main effect of ‘HANDLE’ on RTs [F(4,44) = 8.86; MSE = 0.001; η^2^ = 0.45; p = 0.001].

Post-hoc t-tests revealed this was driven by a difference in the presence of a handle. RTs in the move condition with ‘no handle’ were the shortest (see Table 1) compared to all the other conditions. Significant differences in RTs were identified between the conditions ‘handle on cup’ and ‘handle only’ (both ‘drink’ conditions) versus the ‘no handle’ condition (t(11) = 5.5, p = 0.002 and t(11) = −7.4, p = 0.00013, respectively).

Taken together, these results showed that RTs for large targets, in the absence of a handle, were shorter, whereas RTs in the drink conditions were longer. This reflects speed-accuracy trade-offs previously described when grasping an object with smaller width^39^.

A further ANOVA was performed for the movement times, defined as the time between movement onset and movement offset (MTs, see Methods section), which were entered in a similar one-way ANOVA with 5 conditions for ‘HANDLE’. This revealed no significant main effect (F(1.45,15.9) = 2.4; MSE = 0.004; η^2^ = 0.18; p = 0.13), suggesting no difference in MTs between conditions.

## Discussion

In this study, the kinematic measures of grasp (namely grip aperture) were modulated by the presence of a handle on a cup. The maximal grip aperture (MGA) was smaller when grasping a cup with a handle, compared to grasping a cup with no handle, despite the fact both objects were of the same size at their grasping points (specifically, they had the same height, weight and diameter, see Methods). This effect was observed in the ‘handle toward’, ‘move’ condition, where it was present early on and prior to reaching maximum grip aperture (from the time the grip aperture was at 60% of MGA), when reaching to grasp the cup. To our surprise, there was also an effect of the handle in the ‘handle away’ compared to the ‘no handle’, ‘move’ conditions, even though the handle in this condition was oriented away from the grasping hand. This effect was observed at the time of maximal grip aperture.

Participants had been instructed that in the ‘move’ trials they had to grasp the cup from its rim, between their thumb and index finger, to move it to a location behind. There was no requirement to grasp the cup from its handle on those conditions. There was no significant difference in errors according to trial type, suggesting that participants understood and followed this instruction accurately.

The presence of a handle in the ‘drink’ conditions compared to ‘move’ condition with ‘no handle’ revealed longer reaction times. Previous studies, have reported longer reaction times when grasping an object with smaller width^39^. The effects identified in MGA and RTs suggest that the handle on the cup afforded the action to grasp the cup from the handle, in addition to the action instructed by the task, which required a larger precision grip.

Further analyses investigating the effects of the previous trial on the grip aperture in the next one demonstrated that the affordance effects we identified were not a consequence of carry-over effects.

Taken together, these findings support our hypothesis that execution of the grasp in the ‘move’ conditions likely incorporated the representation of a smaller grip targeted to its handle, when this was present on the cup. Interestingly, by using kinematic measures (namely grip apertures) we were able to obtain an insight into how these processes evolve dynamically over time, in a way that has not been previously described in the literature. We discuss our findings in light of previous literature on the effects of affordances on action, with a particular focus to paradigms eliciting affordances implicitly, comparing these to stimulus-distractor paradigms in which they may be elicited more explicitly in order to make a choice between two actions. We end our discussion with some literature on the cognitive control of affordances, which may modulate their effects.

### Implicit representations of grasp actions triggered by affordances

There are several accounts in the literature on the influence of affordances on action representations (see Introduction)^1–6,12,13,17^. Studies have argued affordances influence movement preparation in reaching tasks, possibly by eliciting motor representations of target locations^10,11,40^. Our results extend these findings and suggest that presence of salient, graspable features on a real everyday object also influence grasping, and that they can be identified during movement execution – quantified here by the grasp aperture profile taken at MGA and earlier time points.

They concur both with recent results demonstrating affordance effects in reaching tasks^40,41^, and with the original study by Riddoch *et al*., which had demonstrated an effect of object handle on the choice of grasp in a patient^36^. Similar results have also been obtained with studies investigating kinematic changes in grip aperture in the context of distractor-target paradigms^11–14,42^. The main difference between our study and these latter paradigms, was that our affordance effects are unlikely to be attributable to an explicit process of competition between actions^19^. As in original studies of affordance^2–6^ the effects of the handle of the cup on grasp representations was observed in the ‘move’ conditions, despite the fact participants were not explicitly required to grasp the handle itself. Even though our task did not explicitly invoke a choice between actions, both small and large precision grips were randomly elicited in response to the instruction and object to use, in the same way as both left and right button presses were required for performance of Tucker and Ellis’ original task demonstrating effects of affordances^2^. Hence our findings suggest that movement representations to grip the handle might have been elicited implicitly and in the absence of a requirement for their use^2,6,17^. Conversely, stimulus-distractor studies such as the ones described by Castiello *et al*.^11–14^ demonstrated interference of grip apertures to target objects when distractor objects of similar size versus different sizes where positioned in near, as opposed to distant locations, suggesting these representations occurred in the context of choice or (‘forced choice’^11^) action selection.

These effects might represent a mechanism by which participants could reprogram their grasp actions in response to changes in the environment. Such processes have recently been shown to also occur implicitly^43^. This mechanism would allow them to adjust their grasp in response to contextual factors and to adapt their choice of grasp to objects of different sizes, shape and orientation.

### Some unexpected findings

It is noteworthy that we observed an affordance effect even when the handle was oriented in the direction opposite to that of the grasping hand. This supports our conjecture that the handle may be triggering a motor representation implicitly to form a small precision grip in this condition, even if this would have led to an uncomfortable grasp posture. Affordance effects have recently been shown to interact with factors affecting biomechanical constraints during grasp actions, measured, for example, with the end state comfort effect^44,45^. These studies suggest that although both affordances and end comfort aim to reduce the manifold of possible actions that can be elicited by providing perceptual or biomechanical constraints, respectively, they are indeed separable and interact with one another such that affordances may, in some instances, override biomechanical constraints to facilitate movement selection under some task conditions^44^.

In our study, we interspersed trials in which participants performed a larger precision grip to the rim of the cup in order to ‘move’ it with trials in which participants performed a precision grip either targeted to the handle of the cup or to a handle on its own. However, unlike the modulation in grip aperture we observed in response to the presence of a handle on the cup in the ‘move’ conditions, there was no modulation of the cup on the handle in the ‘drink’ condition.

One possibility for this finding may be that the grip apertures for the precision grips to the handle in the ‘drink’ conditions were too small to identify subtle differences between conditions using the kinematic measures we opted for, despite the fact it might have been present. Another possibility is that the precision grip performed on the handle in order to ‘drink’ from the cup may be more salient than the action to move it using a larger precision grip to its rim. This would entail that in addition to the affordance effect induced by object features, there might have been a semantic effect provided by the knowledge of the object and its function that reinforces its affordance^42,46–51^. If this is the case, our findings might suggest that participants would consider the affordance of grasping the cup from its body to a lesser extent than grasping it by its handle. Further investigations, using these paradigms in a functional neuroimaging environment, might help identify the underlying mechanisms for these.

### The cognitive control of actions elicited by affordances

Several accounts have, as in our results, identified motoric representations driven by affordances, which were independent of task instruction^2–10^. Although most of them argue for their automatic elicitation, there is evidence to suggest that affordance effects can also be contextually driven^6,17,20,52^.

Original studies suggesting that actions could be triggered ‘automatically’ derive from the neuropsychology literature, where in some conditions affecting inhibitory control, perception may, indeed, *automatically* influence action performance. An example of this is in patients with frontal lobe lesions, who display ‘utilization behaviour’^53^. Similarly, patients with ‘Alien-limb syndrome’ demonstrate an inability to suppress actions elicited by objects or the environment^36,54–56^. However, more recent accounts of these behaviours have challenged this assumption in patient populations, suggesting that the perceptual effects observed on these patients’ actions may depend on the observer’s intentional set and task requirements^6,17,54^. For example, in the study by Riddoch *et al*.^36^, the finding of object-directed actions in a CBD patient was specific to actions that involved grasping of the cup, as opposed to actions involving pointing to it. In addition, they were attenuated when the cup was presented upside down. These results agree with more recent studies in which TMS was used to elicit motoric representations triggered by affordance under conditions of high versus low working memory load. These studies have demonstrated that affordance effects are likely under a form of cognitive control^6,17,57^, possibly involving dopaminergic systems in the brain^58^.

In conclusion, this study is the first to suggest an influence of ‘affordance’ during grasp actions, using kinematic measures. The affordance effects observed here were sustained throughout the reaching phase when grasping an object, and despite there not being a requirement from the task to use an alternative grasp. This extends findings from previous studies, which demonstrated affordance effects using reaction time differences during movement planning^2–10^, by providing evidence that this effect is also present during movement execution. Further studies will be required to investigate the nature of these effects and whether different responses to affordances in grasp may be sub-served by different brain networks involving dorsal and ventral stream pathways^21–26,42,48–51^.

## Methods

### Participants

Twelve right-handed participants aged 23-35 years old (mean age 27.5; 6 Males, 6 Females) with normal or corrected-to-normal vision participated in this experiment after providing informed consent, based on previous studies which used a similar number of participants^19,26^. Eight participants were tested at KU Leuven and 4 additional participants underwent the same procedure at the University of Oxford. The experimental procedures were approved by the ethics committee at KU Leuven and the University of Oxford (Central University Research Ethics Committee), respectively, and were carried out in accordance with the approved guidelines.

### Stimuli

The experimental set-up is outlined in Fig. 1. Participants sat in front of a table on which an object was positioned at a distance of 40 cm in front of them (distance from the edge of the table to object in the midline). The object was placed in a position located centrally, over an area measuring 16 cm^2^ on the table in front of the participant. Behind this was a rectangular cardboard box measuring 10 cm × 15 cm and 3 cm in height, upon which the participants had to place the object when they heard the instruction to ‘move’ it.

One of three objects were presented to participants (see Fig. 1b): a cup with no handle measuring 6.5 cm in height, 4.5 cm in width at the opening and 80 g in weight; a cup with handle of identical dimensions (the handle diameter was 2.5 cm), height of 6.5 cm and width of 4.5 cm at the opening, also weighing 80 g; and a handle made up of a stick and tape measuring 6.5 cm in height and 2.5 cm in diameter (i.e., identical dimensions to the handle of the cup) and weighing 10 g. The cup with a handle was either presented with the handle pointing to the right or the left side. The former (right side) was in the same direction as the grasping hand.

One of two verbal instructions was provided (Fig. 1b and video – supplementary material): either to ‘move’ the object or to ‘drink’ from the object. In the ‘move’ condition, participants had to perform a large precision grip to pick the cup from its rim, between their index finger and their thumb, in order to position it on the box located immediately behind the object. In the ‘drink’ condition they had to perform a small precision grip to pick the handle (only) or the handle of the cup and bring it to their mouth, as if to drink from the object.

### Apparatus

Participants wore liquid-crystal PLATO-occlusion glasses (Milgram, 1987) that allowed the timely start and suppression of vision during the experiment. An electromagnetic tracking system was used to obtain kinematic measures of grasp actions in this task (trakSTAR [NDI Europe, GMbH], sampling rate 240 Hz). Two sensors were used to measure grasp aperture and hand direction. These were positioned 1) on the thumbnail, and 2) the nail of the index finger (Fig. 1a). Informed consent has been obtained from the healthy participant who took part in this experiment, and is depicted in Fig. 1a, to be photographed and filmed during performance of the task and for this to be made available in an online open-access publication.

### Procedure

Participants rested their right hand on the side (against a foam located 20 cm from the midline) with their fingers positioned in a pincer grip (closed). A trial started with the instruction provided by the experimenter. This occurred at the same time as the experimenter pressing a computer key, which triggered the opening of the liquid crystal occlusion glasses, allowing visualisation of the object.

They reached to grasp one of three objects: a cup with a handle, a cup with no handle or a handle only. There were two possible actions they were instructed to make: either to ‘move’ or to ‘drink’. The conditions to ‘move’ were only presented with the cup with a handle pointing to the right (handle towards condition) or the left (handle away) or with the cup with no handle (no handle). In these conditions, participants had to move the cup from a central position to a box located behind, by grasping the object between their thumb and index finger from the top. In the ‘drink’ conditions, which were only presented for the cup with a handle pointing to the right (i.e. the same direction and the grasping hand; handle on cup condition) or for the handle on its own (handle only condition), participants had to grasp the cup from the handle (or the handle by itself) with a pincer grip, in order to bring it to their mouth.

The glasses remained open for 5 seconds. Participants were allowed to perform the instructed action at their own pace. The glasses closed allowing the next trial instruction to be given by the experimenter. Kinematic measures of their grasp were obtained from the start of the movement to the time they grasped the object.

The objects and instructions were presented in random order; the equipment was controlled by a computer program using code written in MATLAB (MathWorks). A total of 100 trials (20 repetitions of each condition) were obtained for each participant.

### Analysis

Kinematics were analysed off-line using custom-written scripts in MATLAB (MathWorks). Maximum grip aperture (MGA) was measured as this occurs before any interaction with the object takes place and is therefore independent of the exact object dimensions, thus providing a good indication of the influence of perception on action^17^. The MGA was calculated as the maximum distance between the thumb and index fingers prior to the object being grasped.

Movement *onset* was defined as the point one of the fingers reached a velocity 120 mm/s that continued to increase to at least 300 mm/s to account for initial noise from small movements of the hand before initiating the grasp. Movement *offset* was the point after the reach, where both fingers’ velocities dropped below 120 mm/s again, which occurred at the time the object was being grasped. Before calculating the velocity, the position data was filtered with a bidirectional low-pass Butterworth filter (second-order, 15 Hz cut-off frequency).

For comparison of the reach traces across trials, movements were aligned in time at the start of the movement and the time point when participants’ MGA was reached. The traces were resampled in 10% steps that reflected the percentage of time to MGA (where 0 was the movement onset and 100% was time of MGA – Fig. 2a and b).

For data analysis, repeated measures Analysis of Variance (ANOVAs) were used to analyse the following dependent variables: MGA, Reaction Times (RTs), Movement Times (MTs), and error rates. All analyses were initially performed using a one-way ANOVA with five levels (HANDLE, which included the 3 ‘move’ conditions and the 2 ‘drink’ conditions). The data were normally distributed, however in some of these ANOVAs (namely MGA), the assumption of sphericity (Mauchly’s test) was violated, so a Greenhouse-Geisser correction was applied to test for statistical significance. All ANOVAs presented here were performed using IBM SPSS Statistic 22 for Windows software (SPSS Inc., Chicago, IL). We used a significance level of 𝛼 = 0.05 both for the kinematic, reaction and movement time and error rate analyses. An additional 2 (previous grasp, large or small) × 5 (HANDLE) ANOVA was performed for MGA to investigate the possibility of carry-over effects from the previous trial.

Moreover, in the presence of a statistically significant effect, post-hoc, paired samples t-tests were performed comparing differences in mean MGA between respective conditions, with a Bonferroni correction.

To further investigate effects of interest, namely differences in MGA over time, we also carried out a comparison on the ‘move’ conditions at different times relative to MGA in a 10 (% MGA step) × 3 (HANDLE) repeated measures ANOVA. The % MGA steps were here the grip apertures at 10–100% of MGA time (Fig. 2b).

## Electronic supplementary material

Drink handle on cup

Drink handle onlly

Move No Handle

Move Handle Toward

Move Handle Away

Supplementary Information

## References

[1] Gibson J. J. *The ecological approach to visual perception* (Boston ed., Houghton-Miffin (1979).

[2] Tucker M, Ellis R (1998). On the relations between seen objects and components of potential actions. Journal of Experimental Psychology: Human Perception and Performance.

[3] Ellis R, Tucker M (2000). Micro-affordance: the potentiation of components of action by seen objects. British Journal of Psychology.

[4] Craighero L, Fadiga L, Rizzolatti G, Umilta C (1999). Action for perception: a motor-visual attentional effect. JEP Human Perception and Performance.

[5] Symes ETM, Ellis R, Vainio L, Ottoboni G (2008). Grasp preparation improves change detection for congruent objects. JEP Human Perception and Performance.

[6] Bub DN, Masson MEJ (2010). Grasping beer mugs: On the dynamics of alignment effects induced by handled objects. JEP: Human Perception and Performance.

[7] Phillips JC, Ward R (2002). S-R correspondence effects of irrelevant visual affordance: time course and specificity of response activation. Visual Cognition.

[8] Kornblum S, Lee J-W (1995). Simulus-response compatibiliy with relevant and irrelevant stimulus dimensions that do and do not overlap with response. JEP: Human Perception and Performance.

[9] Riggio L (2008). The role of attention in the occurrence of the affordance effect. Acta Psychol (Amst).

[10] Tipper SP, Lortie C, Bayliss GC (1992). Selective reaching: evidence for action-centered attention. JEP: Human Perception and Performance.

[11] Castiello U, Bennett KM, Stelmach GE (1993). Reach to grasp: the natural response to perturbation of object size. Experimental Brain Research.

[12] Castiello U (1996). Grasping a fruit: selection for action. JEP: Human Perception and Performance.

[13] Castiello U (1998). Attentional coding for three-dimensional objects and two-dimensional shapes. Differential interference effects. Experimental Brain Research.

[14] Castiello U, Bennett K, Chambers H (1998). Reach to grasp: the response to a simultaneous perturbation of object position and size. Experimental Brain Research.

[15] Corbetta M, Shulman GL (2002). Control of goal-directed and stimulus-driven attention in the brain. Nature Reviews Neuroscience.

[16] Riggio L (2008). The role of attention in the occurrence of the affordance effect. Acta Psychol (Amst).

[17] Masson MEJ, Bub DN, Breuer AT (2011). Priming of reach and grasp actions by handled objects. Journal of Experimental Psychology: Human Perception and Performance.

[18] Cisek P, Kalaska JF (2005). Neural correlates of reaching decisions in dorsal premotor cortex: specifications of multiple direction choices and final selection of action. Neuron.

[19] Cisek P (2007). Cortical mechanisms for action selection: the affordance competition hypothesis. Phil Trans Royal Soc London B Biol Sci.

[20] Young G (2006). Are different affordances subserved by different neural pathways?. Brain Cogn.

[21] Fagg AH, Arbib MA (1998). Modeling parietal-premtor interactions in primate control of grasping. Neural Networks.

[22] Jeannerod M, Arbib MA, Rizzolatti G, Sakata H (1995). Grasping objects: the cortical mechanisms of visuomotor transformation. Trends Neurosci.

[23] Rizzolatti G, Matelli M (2003). Two different streams form the dorsal visual system: anatomy and functions. Experimental Brain Research.

[24] Fattori P (2010). The dorsomedial pathway is not just for reaching: grasping neurons in the medial parieto-occipital cortex of the macaque monkey. Journal of Neuroscience.

[25] Grol MJ (2007). Parieto-frontal connectivity during visually guided grasping. Journal of Neuroscience..

[26] Verhagen L, Dijkerman HC, Grol MJ, Toni I (2008). Perceptuo-motor interactions during prehension movements. Journal of Neuroscience.

[27] Grafton ST, Fadiga L, Arbib MA, Rizzolatti G (1997). Premotor cortex activation during observation and naming of familiar tools. Neuroimage.

[28] Chao LL, Martin A (2000). Representation of manipulable man-made objects in the dorsal stream. Neuroimage.

[29] Grezes J, Tucker M, Armony J, Ellis R, Passingham RE (2003). Objects automatically potentiate action: an fMRI study of implicit processing. Eur J Neurosci.

[30] Binkofski F (1999). A fronto-parietal circuit for object manipulation in man: evidence from an fMRI study. European Journal of Neuroscience.

[31] Buccino G, Sato M, Cattaneo L, Roda F, Riggio L (2009). Broken affordances, broken objects: a TMS study. Neuropsychologia.

[32] Franca M (2012). Corticospinal facilitation during observation of graspable objects: a transcranial magnetic stimulation study. PLoS One.

[33] Muthuhkumaraswamy SD, Johnson BW, McNair NA (2004). Mu rhythm modulation during observation of an object-directed grasp. Brain Res Cogn Brain Res.

[34] Proverbio AM, Adorni R, D’Aniello GE (2011). 250ms to code for action affordance during observation of manipulable objects. Neuropsychologia.

[35] Proverbio AM (2012). Tool perception suppresses 10–12 Hz mu rhythm of EEG over somatosensory area. Biol Psych.

[36] Riddoch JM, Edwards MG, Humphreys GW, West R, Heafield T (1998). Visual affordances direct action: neuropsychological evidence from manual interference. Cognitive Neuropsychology.

[37] Humphreys GW, Riddoch JM (2007). How to define an object: evidence from the effects of action on perception and attention. Mind and Language.

[38] Chinellato E, Castiello U, Sartori L (2015). Motor interference in interactive contexts. Frontiers in Psychology.

[39] Bootsma RJ, Marteniuk RG, MacKenzie CL, Zaal FTJM (1994). The speed-accuracy trade-off in manual prehension: effects of movement amplitude, object size and object width on kinematic characteristics. Experimental Brain Research.

[40] Stewart BM, Gallivan JP, Baugh LA, Flanagan JR (2014). Motor, not visual, encoding of potential reach targets. Current Biology.

[41] Gallivan J. P., Barton K. S., Chapman C. S., Wolpert, D. M. & Flanagan, J. R. Action plan co-optimization reveals the parallel encoding of competing reach movements. *Nature communications*, doi:10.1038/ncomms8428 (2015). 10.1038/ncomms8428 PMC4502063 26130029

[42] Gentilucci M, Gangitano M (1998). Influence of automatic word reading on motor control. Eur J Neurosci.

[43] Ocampo B, Al-Janabi S, Finkbeiner M (2015). Direct evidence of cognitive control without perceptual awareness. Psychon Bull Rev.

[44] Herbort O, Butz MV (2011). Habitual and goal-directed factors in (everyday) object handling. Experimental Brain Research.

[45] Rounis E, Zhang Z, Pizzamiglio G, Duta M, Humphreys G (2017). Factors influencing planning of a familiar grasp to an object: what it is to pick a cup. Experimental Brain Research.

[46] Chainay H, Humphreys GW (2002). Neuropsychological evidence for a convergent route model for action. Cognitive Neuropsychology.

[47] Creem SH, Proffitt DR (2001). Grasping objects by their handles: a necessary interaction between cognition and action. Journal of Experimental Psychology: Human Perception and Performance.

[48] Goodale MA, Milner AD (2000). Separate visual pathways for perception and action. Trends in Neuroscience.

[49] Daprati E, Sirigu A (2006). How we interact with objects: learning from brain lesions. Trends Cogn Sci.

[50] van Polanen V, Davare M (2015). Interactions between dorsal and ventral streams for controlling skilled grasp. Neuropsychologia.

[51] Sakreida, K. *et al*. Affordance processing in segregated parieto-frontal dorsal stream sub-pathways. *Neuroscience & Biobehavioral Reviews* **69**, 89–112. 10.1016/j.neubiorev.2016.07.032 27484872

[52] Tipper SP, Paul MA, Hayes AE (2006). Vision-for-action: the effects of object property discrimination and action state on affordance compatibility effects. Psychonomic Bulletin & Review.

[53] Lhermitte F (1983). ‘Utilization behaviour’ and its relation to lesions of the frontal lobes. Brain.

[54] Shallice T, Burgess PW, Schon F, Baxter DW (1989). The origins of utilization behaviour. Brain.

[55] McBride J, Sumner P, Jackson SR, Bajaj N, Husain M (2013). Exaggerated object affordance and absent automatic inhibition in alien limb syndrome. Cortex.

[56] Fisher CM (2000). Alien hand phenomena: a review with the addition of six personal cases. The Canadian Journal of Neurological Sciences.

[57] Freeman SM, Itthipuripat S, Aron A (2016). High working memory load increases intracortical inhibition in primary motor cortex and diminishes the motor affordance effect. The Journal of Neuroscience.

[58] Friston KJ (2012). Dopamine, affordance and active inference. PLOS Computational Biology.

